# Beyond what we expect: The basics and pedagogical implications of test-enhanced learning in contemporary medical education 

**DOI:** 10.30476/JAMP.2020.86165.1233

**Published:** 2022-04

**Authors:** RAJPRASATH. R, MAGI MURGAN, DINESH KUMAR. V

**Affiliations:** 1 Department of Anatomy, Pondicherry Institute of Medical Sciences, Puducherry, India; 2 Department of Anatomy, Jawaharlal Institute of Postgraduate Medical Education and Research, Puducherry, India

Dear Editor,

Since ages, the word ‘testing’ is equated or irreversibly used for assessment purposes and mostly confined to measuring the amount of knowledge gained by the students in the stipulated period of curriculum or assessment of whether students perform up to a satisfactory level. However, recent literature ( 1 , 2 ) suggests that testing has implicit pedagogical benefits which extends beyond the preamble of assessment. The ability of testing to promote learning and help in long-term retention of related information is referred as “test- enhanced learning” ( 1 ). As a practical example, students tend to retrieve and recall the questions which have been asked in their grade examinations better than the knowledge which haven’t been questioned. Unfortunately, owing to the undue emphasis on high-stakes judgment oriented summative assessments, other critical pedagogical aspects related to testing are often ignored. 

In an interesting study involving post-graduate residents, it was found that the cohort who were tested repeatedly were found to have greater retention of learned content even six months after the teaching encounter compared to those who studied repeatedly ( 3 ). Test-enhanced learning has a profound importance in medical education, where students need to transfer the knowledge in various contexts to facilitate the diagnostic process. A closely related cognitive axiom is ‘spacing effect’ which refers to the learning potentiation which happens when testing is distributed over time, and it has been proven that knowledge retention is more likely when tests are spaced rather than being massed in a short period ( 4 ).

Although the mechanism by which test-enhanced learning strengthens knowledge retention is still enigmatic, we could attribute it to few plausible factors. In their detailed meta-analysis, *Adesope OO et al*. ( 5 ) have demonstrated a strong positive effect of testing effect. They postulated two significant influencers for testing effect: feedback and retrievability (direct benefits of testing). When presented with questions and corresponding answers, students are provided with either positive feedback when the answer is correct or negative feedback when it is wrong. Retrievability refers to the proportion of successful attempts in which students retrieve the learning content from memory, and this could be measured as the number of correct responses in varied testing conditions. *Barnett and Ceci* ( 6 ) had classified the contexts in which the knowledge/skill needs to be transferred as temporal, spatial, functional, social, and modality. For example, the neuroanatomy learned by the student during the first year should be transferred across years (temporal), helpful either in clinics or images (spatial), make clinical correlation (functional), and irrespective of formats (modality). Test-enhanced learning confers the benefit of transferring knowledge across the above-mentioned contexts. Furthermore, students subjected to ‘testing’ condition often use more than one study strategy and thereby chances of retrieval becomes more. A significant study which compared the effect of retrieval practice using free recall test and elaborative study practice using concept mapping found that students belonging to the former group performed better on final tests ( 7 ). In addition, testing offers indirect benefits in terms of motivational factors, memory encoding, strategic time allocation, and so on. 

In spite of its proven benefits across settings, the concept of test-enhanced learning is highly underutilized in medical education owing to the lack of awareness regarding its pedagogical benefits or biases held by the educators towards assessments or overcrowded curriculum. Nevertheless, educators can adopt possible measures at individual level in order to confer the benefits of test enhanced learning to students. Firstly, instructors need to adopt multiple question formats in weekly formative assessments, and this could vary from free-recall type of questions to hybrid formats testing higher cognitive outcomes. Utilizing multiple formats coupled with increased frequency of retrieval and quality feedback would increase the potentiation of memory ( 8 ). Secondly, educators, especially those who handle students in the pre-clinical years, could utilize programmed instruction format whereby shorter sections of the text is provided followed by test questions and associated feedback ( 9 ). When such programmed instruction unwinds from simple to progressively complex material, the ability to store and recall would be better. This also holds strong when clinical vignettes are used for assessment purposes in preclinical curriculum where it should progress from structural to deep level of conceptual learning. Thirdly, students should be explained about how tests enhance learning and how to perform frequent self-assessments. It has been found that students do not adopt self-testing practices themselves because they often consider the metacognitive requirements associated with testing to be burdensome ( 10 ). Furthermore, the pressure induced by the high-stakes examination on them make them under weigh the low stakes formative assessment and no stakes self-assessment. 

In conclusion, we wish that every educator would consider few things in his/her corresponding settings: a) when and how should test-enhanced learning be used in instructional models? b) What question formats could be used for that purpose? c) How to align it with educational objectives? Our intention is to highlight the fact that frequent retrieval practice is equally important as how students are taught in the classroom. Practising retrieval at regular spacing enables long term retention and helps to transfer the knowledge across different contexts. Thus, we suggest the educators to incorporate the principles of test-enhanced learning in all phases of the iterative process of learning. In conditions where high stakes examination leads to cramming and creates an illusion of successful learning, test-enhanced learning, if utilized in an effective manner, could act as an effective pedagogical catalyst in complex educational systems. 

## Conflict of Interest

None Declared.
